# Artificial Authority: The Promise and Perils of LLM Judges in Healthcare

**DOI:** 10.3390/bioengineering13010108

**Published:** 2026-01-16

**Authors:** Ariana Genovese, Lars Hegstrom, Srinivasagam Prabha, Cesar A. Gomez-Cabello, Syed Ali Haider, Bernardo Collaco, Nadia G. Wood, Antonio Jorge Forte

**Affiliations:** 1 Division of Plastic Surgery, Mayo Clinic, Jacksonville, FL 32224, USA; 2 Center for Digital Health, Mayo Clinic, Rochester, MN 55905, USA; 3 Department of Radiology AI IT, Mayo Clinic, Rochester, MN 55905, USA; 4 Department of Artificial Intelligence and Informatics, Mayo Clinic, Jacksonville, FL 32224, USA

**Keywords:** artificial intelligence, large language model, clinical decision support systems, decision making, computer assisted, benchmarking, LLM-as-a-judge

## Abstract

Background: Large language models (LLMs) are increasingly integrated into clinical documentation, decision support, and patient-facing applications across healthcare, including plastic and reconstructive surgery. Yet, their evaluation remains bottlenecked by costly, time-consuming human review. This has given rise to LLM-as-a-judge, in which LLMs are used to evaluate the outputs of other AI systems. Methods: This review examines LLM-as-a-judge in healthcare with particular attention to judging architectures, validation strategies, and emerging applications. A narrative review of the literature was conducted, synthesizing LLM judge methodologies as well as judging paradigms, including those applied to clinical documentation, medical question-answering systems, and clinical conversation assessment. Results: Across tasks, LLM judges align most closely with clinicians on objective criteria (e.g., factuality, grammaticality, internal consistency), benefit from structured evaluation and chain-of-thought prompting, and can approach or exceed inter-clinician agreement, but remain limited for subjective or affective judgments and by dataset quality and task specificity. Conclusions: The literature indicates that LLM judges can enable efficient, standardized evaluation in controlled settings; however, their appropriate role remains supportive rather than substitutive, and their performance may not generalize to complex plastic surgery environments. Their safe use depends on rigorous human oversight and explicit governance structures.

## 1. Introduction

### 1.1. Background

Trust is the currency of medicine. For centuries, patients have depended on clinicians to deliver accurate information, sound judgment, and compassionate care. Clinicians, in turn, have relied on rigorously vetted knowledge sources, such as training, peer-reviewed research, regulatory oversight, and professional consensus, to ensure that medical decisions reflect the highest standards of evidence and ethics. This carefully constructed chain of validation has long safeguarded patient welfare and upheld confidence in the healthcare system.

Artificial intelligence (AI) now extends and challenges this paradigm. By training on vast medical corpora and general knowledge datasets, large language models (LLMs) can synthesize information at a scale and speed no human can match [1,2]. Large language models are a class of AI systems trained with deep neural networks to generate and interpret text by learning statistical patterns from large-scale datasets [3]. Rather than reasoning from first principles or clinical experience, LLMs operate by estimating the likelihood of sequences of words given a prompt [4], which allows them to produce fluent responses without explicit understanding or intent.

Recent studies demonstrate performance at or near expert level in clinical reasoning, developing differential diagnoses, and giving treatment recommendations [5,6,7,8]. In specialties such as dermatology, ophthalmology, and oncology, models have rivaled, and in constrained tasks occasionally exceeded, clinician accuracy, particularly where success is correlated with rapid pattern recognition or large-scale knowledge retrieval [9,10,11,12]. In plastic and reconstructive surgery, models have demonstrated efficacy in patient education, physician education, and clinical decision-support [13,14,15]. Their strengths reflect the essence of machine capability: broad knowledge exposure, instantaneous recall, and the ability to detect subtle probabilistic relationships invisible to human cognition.

Yet these same systems are prone to critical failure modes that humans are trained to avoid. LLMs can fabricate plausible but false medical facts, apply guidelines without clinical nuance, hallucinate laboratory values or citations, and express unwarranted certainty [13,16]. Unlike clinicians, who are taught to question, qualify, and consult evidence, LLMs may present incorrect conclusions with polished fluency and authoritative tone [17]. They lack lived clinical context, moral accountability, and the experiential judgment that distinguishes competence from wisdom [18,19]. Furthermore, it has been shown that LLM performance may not be adequate for some clinical tasks (e.g., predicting retinopathy of prematurity) without advanced, structured external inputs [20]. In safety-critical environments, such as the operating room, these limitations are not merely technical flaws; they are vectors for patient harm, diagnostic delay, equity gaps, and erosion of trust.

**Figure 1:**
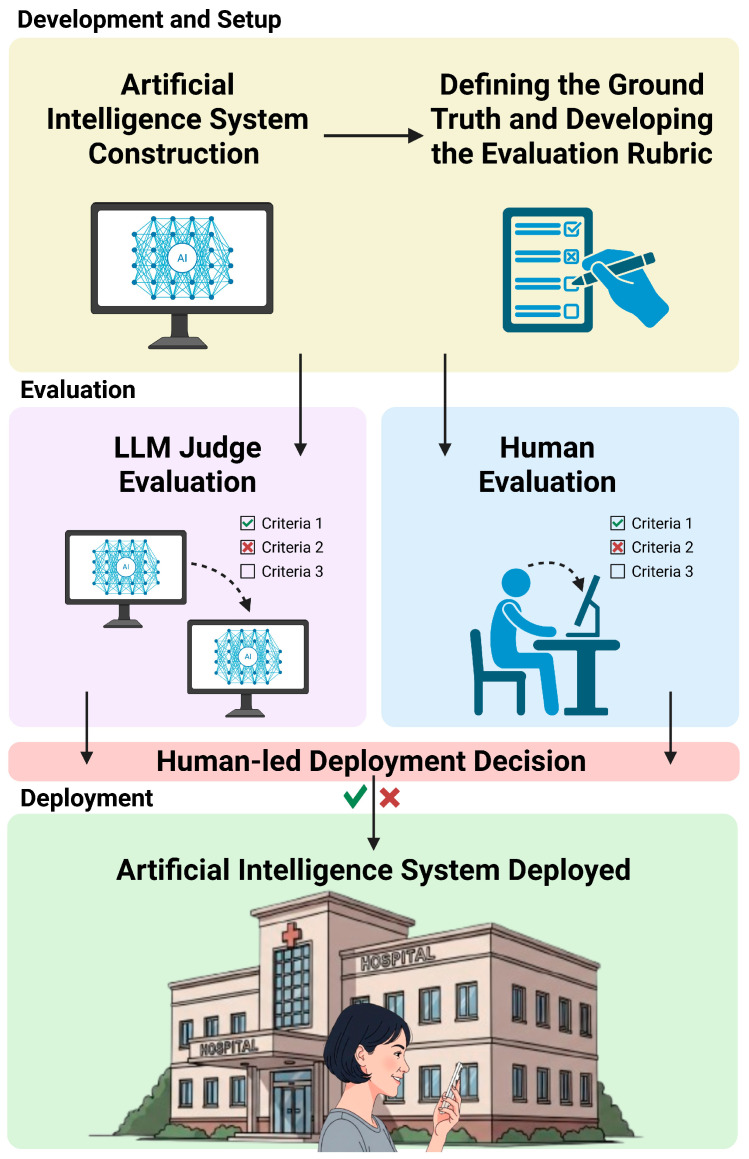
Illustrated is the LLM-as-a-Judge workflow. An artificial intelligence system is developed for a clinical task, after which ground-truth references and an evaluation rubric are defined. System outputs are then assessed by either an LLM-based judge or human reviewers (dashed arrows), informing a final human-led deployment decision (green checkmark or red cross). Created in BioRender. Genovese, A. (2025) [25]. https://biorender.com/ddo2y59, accessed on 16 December 2025.

As LLMs shift from peripheral support functions toward tasks embedded in clinical decision-making, documentation, and patient communication, healthcare finds itself at an inflection point. We are no longer merely asking machines for assistance—we are increasingly asking them for judgment. Compounding this shift, the field has begun adopting a new paradigm known as LLM-as-a-judge, where language models are tasked with evaluating the quality of information produced by other AI systems [21]. For instance, instead of relying on humans or limited automated metrics (e.g., BLEU, ROUGE, BERTScore), an LLM judge processes the AI-generated output (e.g., a clinical summary or recommendation) and then produces an evaluation much like a human reviewer would. This workflow is presented in Figure 1. Furthermore, recent work has highlighted the limitations of human evaluation in this domain: although expert review remains the gold standard, it is constrained by issues of availability and interrater variability, often requiring the use of non-expert reviewers or alternative strategies [22]. LLM-based evaluation offers a compelling alternative—one that promises speed, consistency, and scalability across the expanding ecosystem of AI-assisted clinical and surgical tools [23,24]. If reliable, it could strengthen the chain of trust by enforcing high standards before systems touch patients.

Yet this opportunity brings responsibility, as medicine cannot assume that evaluators without clinical training, situational awareness, or real moral stakes can adequately safeguard care. A model capable of generating a flawed recommendation may also endorse one. Likewise, a system that speaks with confidence may validate confidence where humility is required. In effect, healthcare risks allowing unverified intelligence systems to certify one another—a recursive trust cascade without the anchor of human oversight. Should such judgments be wrong, the consequences extend beyond individual errors: they threaten the legitimacy of AI-enabled medicine and the clinician–patient covenant on which care depends.

### 1.2. Research Objectives

To responsibly harness the promise of LLMs, the field must establish rigorous, clinically grounded frameworks for evaluating their behavior not only for factual accuracy, but for humility, empathy, equity, clarity, and avoidance of harm. This review examines the emerging practice of using LLMs as judges through a clinical and plastic surgery lens and aims to answer the following questions:

1. What methodologies are used to implement LLMs as evaluators?
2. In which clinical application domains have LLM judges been studied, and with what outcomes?
3. How well do LLM-based judges align with human clinicians across different evaluation tasks?
4. What factors influence the performance and reliability of LLM judges?
5. What limitations, risks, and ethical concerns accompany the use of LLMs as evaluators in healthcare?

Accordingly, the review first examines representative LLM-as-a-judge architectures, then surveys their application across key clinical domains, and finally synthesizes cross-study analyses and ethical considerations that bear on their reliability, governance, and appropriate role in clinical practice to strengthen, rather than destabilize, the chain of trust that defines safe and humane medical practice.

## 2. LLM Judge Evaluation Architectures

### 2.1. G-EVAL Method

Liu et al. introduced G-EVAL, a structured evaluation architecture that transforms an LLM into a systematic judge by pairing rubric-based prompting with auto-generated chain-of-thought (CoT) ‘evaluation steps’ and a probability-weighted scoring mechanism [26]. In contrast LLM-evaluators that rely on generation likelihood or unstructured judging, G-EVAL instructed GPT-3.5 or GPT-4 to first produce a reasoning scaffold describing how to apply each evaluation criterion, and then to issue ratings in a form-filling format whose final scores were refined into continuous values using the model’s token-level output probabilities. Evaluated across three meta-evaluation benchmarks—SummEval for summarization, Topical-Chat for dialogue, and QAGS for hallucination detection—G-EVAL-4 achieved the strongest human alignment among all compared metrics, with summary-level Spearman correlations up to 0.514. CoT guidance improved performance, especially on fluency, and probability normalization better captured subtle differences between texts. However, the authors also documented a notable evaluator bias: G-EVAL-4 systematically scored GPT-3.5-generated summaries higher than human-written ones even when human annotators preferred the latter, raising concerns about self-reinforcing reward loops if LLM judges are used in model training. As an evaluation architecture, G-EVAL demonstrated that structured prompting, explicit reasoning scaffolds, and probabilistic scoring can substantially strengthen LLM-as-a-judge reliability, while highlighting persistent vulnerabilities related to prompt sensitivity, model-size dependence, and bias toward LLM-generated text.

### 2.2. LLM-Judge Specialists

Kim et al. tackled a key methodological problem for LLM-as-a-judge systems: most open-source evaluators score differently than humans and struggle to do both direct assessment and pairwise ranking [27]. To address this, the authors developed Prometheus 2, an evaluation architecture that trains one LLM to act as a judge across both major evaluation formats (scoring a single answer and choosing the better of two answers) by merging models trained separately on each task. Using a large and diverse supervision dataset that includes more than 1000 custom evaluation criteria, they benchmarked the system across eight evaluation tasks (4 direct assessment and 4 pairwise ranking) and showed that Prometheus 2 scored high correlations with human evaluators in proprietary LM judges. The method also proved more stable and generalizable than earlier evaluators, performing strongly even on tasks it had not been explicitly trained for. While this study supports that merging language models trained in both evaluations can be reliable when trained on varied evaluation formats, the indirect evaluation method and limited scoring methods (1–5 scoring scale and comparative evaluation) may limit its generalizability and usability.

### 2.3. LLM Jury Method

Verga et al. [28] examined a foundational limitation of LLM-as-a-judge evaluations—namely, that relying on a single large judge (typically GPT-4) introduces cost, instability, and strong intra-model biases—and proposed instead a Panel of LLM Evaluators (PoLL), an ensemble of smaller, heterogeneous models that vote on quality judgments. Across three evaluation settings (single-hop QA, multi-hop QA, and Chatbot Arena head-to-head comparisons) and six datasets, the authors showed that PoLL consistently aligned more strongly with human judgement compared to a single judge. For example, PoLL achieved the highest Cohen’s κ across all single-hop QA datasets (e.g., κ = 0.906 on TriviaQA, surpassing GPT-4’s 0.841) and produced the strongest correlation with human-derived Chatbot Arena rankings (Pearson 0.917, Kendall-τ 0.778), outperforming GPT-4 in both cases. The panel also reduced judge bias: individual judges exhibited large scoring (e.g., standard deviation of 6.1 for ChatGPT-3.5) whereas PoLL produced the smallest error spread (standard deviation of 2.2 for PoLL) in multi-hop QA accuracy differences relative to human judges. PoLL was also far more economical, costing 1.25 USD/input and 4.25 USD/output, compared to GPT-4 Turbo which cost USD 10/input and USD 30/output. The authors note, however, that it is unclear how the model would perform in other settings, such as math and reasoning, which may limit clinical applicability. Collectively, the study demonstrated that diverse multi-model juries can outperform a single “supreme judge” in stability, cost, and bias reduction. Table 1 provides a comparison across the judging architectures reviewed.

## 3. Emerging Applications of LLM-as-a-Judge in Clinical AI

### 3.1. Clinical Summaries and Documentation

One prominent application of LLM judges is in assessing AI-generated clinical summaries of electronic health records (EHRs), allowing clinicians a more efficient, targeted review. Croxford et al. introduced a medical LLM-as-a-Judge framework to evaluate multi-document EHR summaries using a validated rubric called the Provider Documentation Summarization Quality Instrument (PDSQI-9), which defines nine quality attributes (e.g., accuracy, thoroughness, organization, cited) tailored to clinical summaries [29]. In their evaluation, a GPT-o3 mini judge achieved strong agreement with human clinicians, with an intraclass correlation coefficient (ICC) of approximately 0.818 (95% CI 0.772–0.854) and a median score difference of zero compared to experts, suggesting that an LLM can emulate expert judgment on complex clinical documents. Notably, the AI judge completed each evaluation in 22 s, whereas physicians took around 10 min per summary. The study also experimented with various prompting and training strategies from zero-shot and few-shot prompts to fine-tuning and multi-agent (committee) approaches. Zero-shot prompting supplies only instructions, one-shot provides a single example, and few-shot prompting provides multiple examples to teach the model the expected structure and reasoning style [30]. Few-shot degraded performance for GPT-4o and other models, underscoring that the value of example-based prompting for LLM judges is strongly model-dependent. They also found that those employing chain-of-thought prompting or instruction tuning achieved the highest reliability, outperforming both simpler prompts and multi-LLM “jury” frameworks [29].

Expanding on EHR-based LLM judges, Chung et al. [31] introduced VeriFact, a system that couples retrieval-augmented generation, a method of grounding LLM responses in a source to reduce hallucinations [13], with an LLM-as-a-judge to verify long-form clinical narratives. The authors [31] selected 100 patients from a freely available de-identified EHR dataset whose discharge summaries contained a Brief Hospital Course (BHC). For each patient they extracted the clinician-written BHC and generated a de novo BHC via summarization, then decomposed each text into propositions (sentences or LLM-extracted atomic claims) that were annotated by three clinicians. Inter-clinician agreement ranged from 88.5% for LLM-generated atomic claims to 66.6% for human-written sentence propositions and annotating all 13,290 propositions required 1618 h of clinician time. VeriFact’s best-performing configuration achieved 92.7% agreement with the clinician ground truth on sentence propositions and 88.8% on atomic claims, with performance improving as more facts were retrieved and more advanced search methods were used. The study notes that the system often labels human-written statements as “Not Supported,” reflecting information asymmetry when clinicians reference details absent from the EHR. It also reports that sentence-level propositions yield higher agreement but produce a much larger proportion of invalid statements (19.8%) than atomic claims (0.4%). Overall, VeriFact demonstrated that RAG-enabled LLM judges can match or exceed average clinician performance on patient-specific fact-checking while exposing challenges in calibration and handling of negative labels.

Brake and Schaaf [32] extend the use of LLM-as-a-judge into documentation by assessing the internal consistency of automatically generated sections of SOAP notes. Standard automatic metrics (ROUGE and a PEGASUS-trained factuality score) were unable to differentiate between two distinct PEGASUS-X architectures—one generating whole multi-section notes (GENMOD) and one generating each section independently (SPECMOD)—despite clear discrepancies in internal consistency detectable by humans. Their human evaluation (3 medical experts, 2 non-experts) of 40 SOAP-style notes revealed that GENMOD produced substantially fewer contradictions in age, gender, and body-part references than SPECMOD, even though both models scored nearly identically on automated benchmarks. To test whether an LLM could replicate these nuanced human judgments, the authors evaluated Llama-2-70B across zero-, one-, and few-shot prompting strategies, finding that the LLM achieved Cohen’s κ values comparable to or exceeding those of individual human reviewers on age (κ = 0.79), gender (κ = 1.00), and to a lesser degree anatomical consistency (κ = 0.32) for two-shot prompting, and reviewers themselves showed variation in interpreting “coherence.” As such, this study adds complementary insight to the emerging literature: LLM judges are not only useful for scoring correctness but can function as a scalable proxy for detecting subtle, intra-document inconsistencies that escape both automated metrics and manual review at scale, while also underscoring the need for clearer rubric definitions and careful oversight when using LLMs in evaluative roles.

### 3.2. Medical Question-Answering

Another important domain in which LLM judges are gaining traction is the evaluation of medical question-answering, historically dependent on human assessors to determine the quality and safety of system outputs. Recent work [21] presents a systematically constructed framework for employing large language models as automated evaluators of medical these systems, illustrating how LLM-based adjudication can approximate and sometimes exceed clinician review. Using a clinician-curated dataset of 94 assessment sets that included medical questions, ground-truth answers, and in-house system-generated outputs, the authors developed a six-dimension scoring rubric (relevance, succinctness, medical accuracy, hallucination, completeness, and coherence) and iteratively refined a GPT-4o-based judging prompt through the incorporation of expert-annotated examples and domain-specific scoring guidelines. Across these iterations, overall mean absolute error decreased substantially (overall error from 1.20 to 0.94 and 0.62 on a 0–3 scale, for initial prompt, prompt with examples, and prompt with guidelines, respectively). Qualitative auditing revealed that the LLM was capable of catching subtle clinical inaccuracies missed by human reviewers such as incorrect inclusion of non-asthma medications, suggesting that LLM-as-a-judge systems may provide a valuable layer of secondary validation for safety-critical domains. The reported reduction in evaluation time (from ~6 h of clinician effort to about 35 min with LLM assistance) further underscores the operational impact of automated evaluators, though the authors emphasize limitations including modest sample size, single-reviewer comparison, dataset bias, and the need for multi-model ensembles and broader clinical benchmarks.

Diekmann et al. [33] investigated LLM-as-a-judge in the safety-critical setting of patient-facing medical question answering by benchmarking how well open-source LLM “safety judges” aligned with clinicians across a structured set of qualitative risk dimensions. Using 270 patient-facing QA pairs from the CDC subset of MedQuAD, each answered by four generator models (1080 responses total), they obtained three independent expert annotations per answer on eight metrics (Scientific Consensus, Inappropriate/Incorrect Content, Missing Content, Extent of Possible Harm, Likelihood of Possible Harm, Bias, Empathy, and Grammaticality) using 2–3 level categorical scales (e.g., Scientific Consensus: No Consensus/Opposed/Aligned) and took the median label as ground truth. Inter-annotator agreement was high at the label level (95.8% of responses had at least two annotators agree; 66.3% full agreement) but Krippendorff’s alpha values were modest due to highly skewed class distributions, particularly for rare “high-severity” categories. Five LLMs (Meta-Llama-3-70B-Instruct, Llama3-OpenBioLLM-70B, Prometheus-2, Llama3-Med42-8B, Mixtral-8x7B-Instruct) were then prompted as evaluators, each assigning one categorical label per metric per answer, with prompt design iteratively refined to ensure valid outputs. Across metrics, the models showed very high accuracy on relatively objective dimensions—Scientific Consensus (mean 0.976), Bias (0.966), and Grammaticality (0.990)—and strong performance on Inappropriate Content (0.954), but substantially weaker and more variable alignment on Missing Content (mean 0.682, with Mixtral as low as 0.13), Extent of Harm (0.700), Likelihood of Harm (0.786), and especially Empathy (mean 0.486). To probe robustness, the authors also generated 35 adversarial QA pairs whose answers were deliberately “Opposed to Consensus” yet superficially plausible; all models correctly flagged almost all of these as high-severity for Scientific Consensus, with only a single failure involving a subtle misstatement about Kyasanur Forest Disease transmission.

### 3.3. Clinical Conversation Evaluation

Providing insight on LLM judges in assessing interaction patterns within clinical encounters, Li et al. [34] examined whether large language models can reliably score the quality of therapeutic relationships in real, online text-based psychotherapy by using them as judges of the working alliance: counselors’ and clients’ agreement on goals, collaboration on therapeutic tasks, and the affective bond between them. To ground LLM judgments in clinical theory, the authors adapted the observer-rated Working Alliance Inventory-Short form (WAI-O-S) to text-only transcripts and developed behaviorally anchored scoring guidelines. On a corpus of 859 sessions between 9 counselors and 82 clients, they then evaluated whether four LLMs (GLM-4, Claude-3, ChatGPT, GPT-4) can rate each conversation on the same 1–5 scale. They also added chain-of-thought (CoT) evidence extraction for further assessment. Across conditions, model self-consistency (ICC) was moderate to high for GLM-4 and Claude-3 and lower for ChatGPT, while alignment with human ratings, measured by Pearson correlation, improves markedly with richer guidance: detailed guidelines raise average correlations by 23.61%, and for a particularly subtle “liking each other” item (Q9), GPT-4’s correlation increases by 76% relative to minimal guidance. The best configuration (GPT-4 with detailed guidelines + CoT) reaches an overall correlation of approximately 0.50 with human experts across Goal, Approach, and Bond. In a small proof-of-concept, two counselors who struggled with relationship-building rated the LLM’s narrative feedback as modestly helpful (mean ratings 3.43–3.74 on three 5-point items).

Zheng et al. [35] extended the LLM-as-a-judge paradigm into conversational medical education by framing the model as a “fuzzy” clinical evaluator of students’ communication in LLM-simulated patient encounters. Working within the 2-Sigma educational platform, they collected 2302 utterances from medical students interacting with AI patients and had seven expert judges label each utterance along four fuzzy, gradated criteria—Professionalism (three levels), Medical Relevance (three), Ethical Behavior (five), and Contextual Distraction (four)—thereby encoding human preferences about what constituted appropriate, relevant, and ethically sound clinical dialogue in a more nuanced way than pass/fail scoring. They fine-tuned several text-focused backbones (e.g., ClinicalBERT, DeBERTa-v3, LLaMA-3.1-8B, Mistral-7B) in a multi-task setup and combined this supervised fine-tuning with few-shot prompt engineering at inference time, creating a hybrid LLM-as-a-Fuzzy-Judge that predicted fuzzy labels for all four dimensions from each student message. On a held-out test set, the best hybrid model achieved accuracy of 0.84 for Professionalism, 0.82 for Medical Relevance, 0.82 for Ethical Behavior, and 0.83 for Contextual Distraction, with weighted F1-scores around 0.81–0.84, consistently outperforming both a prompt-only LLM and a majority-class baseline and exceeding 80% accuracy across all criteria. Qualitative analysis of borderline and ethically sensitive cases suggested that the model could mirror human-like gradations. For example, labeling inputs as “questionable” rather than fully “safe” or “unsafe,” and assigning high-confidence fuzzy judgments even in ambiguous contexts. The authors noted limitations such as underlying model biases and variability output from prompt engineering.

## 4. Cross-Study Thematic Analysis

### 4.1. LLM Judges Are Being Used to Operationalize a Wide Range of Constructs, from Factual Correctness to Interaction Quality

Across studies, LLMs are used as evaluators of varying targets: medical QA quality [21,33], EHR summaries and narratives [29,31], internal consistency of SOAP-style notes [32], understanding provider–patient relationships [34], and medical student communication behaviors via fuzzy criteria such as professionalism and ethical behavior [35].

### 4.2. LLM Judges May Better Align with Clinicians on Concrete, Observable Dimensions than on Subjective or Affective Ones

A consistent pattern identified through this review is that LLM Judges reach high agreement with clinicians for relatively objective criteria such as scientific consensus, grammaticality, internal consistency, and factual support, but show reduced alignment for empathy, perceived harm, or subtle relational qualities. Diekmann et al. report high LLM accuracy on consensus and grammaticality but substantially lower agreement on empathy and harm severity [33], while Li et al. show that even with detailed guidelines and CoT prompting, GPT-4 only achieves moderate correlations (0.4667) with human evaluation on affective bond items in counseling [34]. Together, these results suggest that current LLM judges are better suited for detecting concrete errors and inconsistencies than for replacing human judgment on nuanced clinical or relational assessments; for fields such as plastic and reconstructive surgery, where documentation, perioperative decision rationale, and postoperative communication span both objective and subjective dimensions, this may point to an appropriate role for LLM judges in structured quality checks rather than higher-order interpretive evaluation.

### 4.3. Judge Performance May Increase When the Evaluation Is Structured

Evidence-grounded evaluation architectures demonstrated that LLM judges align more closely with clinicians when asked to evaluate atomic claims or retrieved clinical evidence instead of long-form narratives. VeriFact’s RAG-based fact checking [31] and Brake and Schaaf’s evaluation of internal consistency in sectioned clinical notes [32] showed that proposition-level or cue-focused inputs improved accuracy in detecting contradictions, and unsupported assertions. Zheng et al. further encoded communication quality through fuzzy sets (e.g., Medical Relevance, Ethical Behavior) and fine-tuned an LLM-as-a-fuzzy-judge that achieves >80–90% accuracy [35]. These designs suggest that decomposing narratives into smaller, distinct evaluation areas can improve LLM judgment quality, an approach that may be particularly advantageous in surgical applications, where operative notes, perioperative plans, and postoperative instructions are highly structured yet interdependent and amenable to discrete assessment.

### 4.4. The Reasoning Process (Advanced Prompt Engineering) Imposed on the LLM May Improve Reliability and Agreement with Human Experts

Across both general and clinical domains, LLM-as-a-judge performance improves with advanced prompting using chain-of-thought. G-EVAL with CoT achieved a higher correlation than without CoT on all domains, providing better guidance and context [26]. In parallel, clinical papers such as Croxford et al. and Li et al. show CoT prompting affect judge–clinician agreement in EHR summarization, and counseling alliance evaluation [29,34]. Beyond these findings, explicit reasoning traces may also enhance transparency by clarifying how individual inputs contribute to assessments, supporting clinician trust and shared decision-making between a surgeon and the patient [36]. This perspective echoes literature in oral and maxillofacial surgery, where encouraging CoT has been associated with improved LLM performance on domain-specific clinical queries [37], underscoring the potential value of transparent reasoning in procedurally complex fields.

### 4.5. LLM Judges Can Often Match Human Evaluations and Exceed Average Clinician Agreement, but Their Performance Is Bounded by Data and Deployment Context

**Figure 2:**
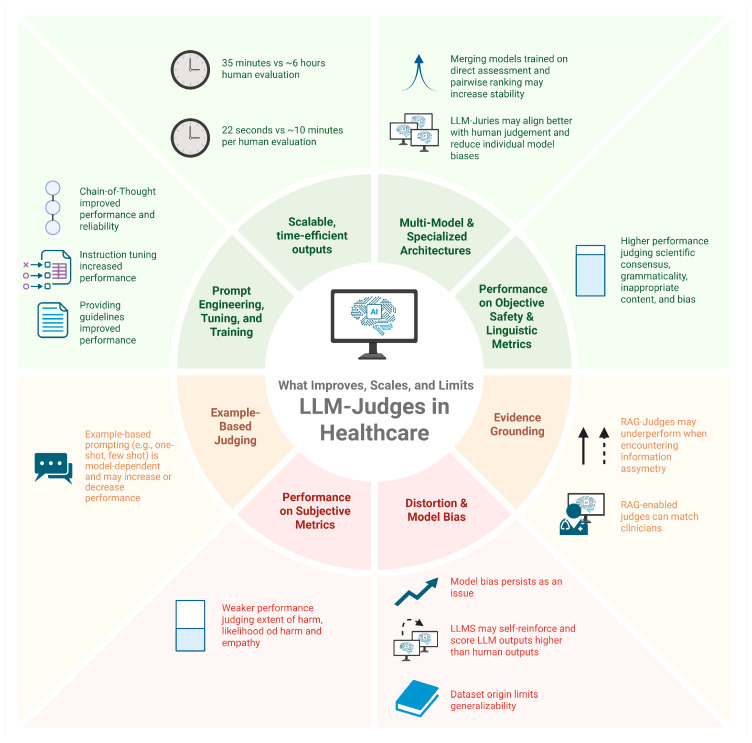
Illustrated is the current performance and risk landscape of LLM-as-a-Judge, with the colors green, yellow, and red indicating positive, neutral, or negative components, respectively. Positive areas reflect scalability, efficiency, and performance on structured or objective evaluation tasks, while mixed and negative regions highlight variability across settings, reduced reliability for subjective judgments, and concerns related to bias and generalizability. Created in BioRender. Genovese, A. (2026) [38]. https://biorender.com/74iqinv, accessed on 1 January 2026.

Across multiple studies, LLM judges could emulate human evaluation [21] and meet or surpass average inter-clinician agreement—for example, VeriFact’s long-form fact checking [31] and Croxford et al.’s GPT-based scoring of clinical summaries [29]. Yet, LLM judges are constrained by the underlying datasets and labels: annotator disagreement on subtle categories (e.g., harm, empathy, alliance strength), class imbalance (e.g., few high-harm examples), and information asymmetry between EHR and human authors limit how “correct” any judge can be [31,33]. Most work is also task-specific, without prospective testing in live clinical workflows, and therefore, cannot be assumed to generalize. The cross-study thematic analysis is depicted visually in Figure 2.

## 5. Discussion

### 5.1. Interpretation of the Results

Across the studies reviewed, the central additive signal is not simply that “LLM judges can work,” but that their apparent reliability is inseparable from how the evaluation problem is operationalized. When tasks are defined around observable properties, LLM judges can approximate clinician ratings because the judgment boundary is relatively explicit and the rubric can be applied reproducibly. The importance of objective judging criteria is echoed by the literature where interactive tools are being developed to define and calibrate such rubrics for LLM-as-a-Judge systems, noting how humans often struggle to articular clear criteria [39]. However, it has been shown that even when a high level of agreement is reached with humans, LLM judges can still struggle with objective criteria, but providing human-authored reference answers combats this [40].

Furthermore, domains requiring clinical, relational inference or counterfactual safety reasoning place heavier demands on contextual understanding and value-based tradeoffs, and the reviewed evidence shows weaker and more variable agreement in these settings. Available evidence suggests a similar pattern where LLM evaluations do not adequately capture contextual nuance [39,40,41,42]. Clinically, this distinction matters because it delineates where LLM judges can credibly function as quality control versus where they risk becoming a veneer of objectivity over inherently interpretive judgments. A key unresolved challenge is how to evaluate constructs such as harm, empathy, or clinical adequacy without oversimplifying them into checklists that fail to reflect real clinical decision-making, as well as meeting the need for evolving criteria in rapidly changing fields such as plastic surgery.

A second implication of these findings concerns how performance of LLM judges is interpreted and compared across studies. Agreement with clinicians is not an intrinsic property of the judge alone but depends heavily on how evaluation labels are defined and distributed. Some of the reviewed studies rely on rubrics with imbalanced categories or vague boundaries between classes; under these conditions, high overall agreement can coexist with systematic blind spots, particularly for infrequent or context-dependent failure modes [33,34]. This creates uncertainty about whether reported performance reflects true safety or merely success on common, low-risk cases, and highlights the need for evaluation designs that explicitly test high-severity and edge-case scenarios. From a clinical perspective, this limits the evidentiary value of aggregate agreement metrics and underscores the need to assess where LLM judges fail, not only where they succeed, before they are used to inform deployment decisions.

### 5.2. Ethical Considerations

The use of LLMs as judges in clinical AI evaluation raises foundational ethical concerns about authority and accountability. Because evaluative judgments—whether about accuracy, safety, or communication quality—shape which models are deployed and how clinicians are assessed, LLM judges must be viewed not as neutral arbiters but as systems that inevitably reflect the biases, omissions, and structural inequities embedded in their training data [43]. This makes governance essential: LLM judges should be developed with transparent, auditable criteria; tested across diverse demographic, linguistic, and clinical settings; and validated against robust, multi-annotator human standards to prevent a single perspective from being encoded as “ground truth.” Without such safeguards, biased evaluations can entrench disparities, penalize clinicians serving marginalized populations, or systematically mischaracterize patient communication that differs from the distributions present in the training corpus [44]. To protect patients and clinicians, the outputs of LLM judges must remain advisory rather than authoritative, with mandated human oversight, external auditing for bias and drift, continuous post-deployment monitoring, and clear mechanisms for appeal or override. Ultimately, ethical deployment requires acknowledging that LLM judges must never displace human judgment, and that their use must be guided by conservative, patient-protective standards that recognize the profound risks of embedding biased automated evaluation into clinical care AI systems.

### 5.3. Strengths and Limitations

While prior reviews have examined the performance and safety of large language models in healthcare, as well as their use as judges in general settings, to our knowledge this is the first review to focus specifically on LLM-as-a-judge architectures in healthcare and their implications for clinical evaluation. Because this is a rapidly evolving methodological area, much of the foundational work currently exists in preprint form, and this review therefore includes both peer-reviewed and non-peer-reviewed studies. This approach is necessary to characterize the current state of the field but should be interpreted with appropriate caution, as the evidence base remains provisional and subject to revision as formal peer review progresses.

### 5.4. Future Directions

Future work on LLM judges in healthcare should move beyond proof-of-concept studies toward a disciplined science of evaluator design, validation, and governance. Methodologically, this means developing domain-specific judge architectures that combine structured rubrics, advanced evidence retrieval, chain-of-thought reasoning, and multi-model “jury” designs, and then stress-testing them across institutions, specialties, and data regimes rather than on single datasets. Empirically, LLM judges need prospective, workflow-embedded assessment that measures how LLM-guided evaluation changes model deployment decisions, clinician behavior, and ultimately patient-relevant outcomes, including equity and access. Governance work is equally important: the field will need shared standards for “approving” an LLM judge (minimum reliability on safety-critical dimensions, required bias audits, subgroup analyses), clear boundaries on acceptable use, and tools for continuous auditing. Finally, because LLM judges will evolve, research should explore systems that periodically re-evaluate and recalibrate judges against updated human benchmarks.

## 6. Conclusions

LLM-as-a-judge systems offer a promising path toward scalable, reproducible evaluation of clinical AI, but their reliability depends entirely on the rigor of their design—clear rubrics, structured reasoning, evidence grounding, and careful validation. Across tasks, LLM judges can approximate human agreement on well-defined criteria, yet they remain vulnerable to biases, data limitations, and contextual blind spots that preclude their use as authoritative arbiters in healthcare. Their role must therefore be constrained to augmenting and perhaps enhancing human evaluation rather than replacing it, with strong safeguards, transparent auditing, and continuous monitoring to ensure that automated evaluation strengthens, rather than quietly erodes, the chain of trust on which clinical AI must rest.

## Figures and Tables

**Table 1:** Comparison of Representative LLM-as-a-Judge Architectures.

Dimension	G-EVAL	LLM-Judge Specialists (Prometheus 2)	LLM Jury Method (PoLL)
Judging Architecture	Single LLM evaluator using form-filling paradigm, auto-generated chain-of-thought, and probability-weighted scoring	Single open-source LLM evaluator trained via weight merging of direct-assessment and pairwise-ranking models	Small heterogeneous panel of LLM evaluators (instantiated with three models from different families)
Target of Evaluation	NLG output quality across summarization, dialogue generation	General language generation quality using user-defined evaluation criteria	Question answering and conversational model outputs
Strengths	Structured evaluation, improved human alignment, fine-grained continuous scoring	Unified evaluation across formats, open-source and reproducible	Reduced evaluator bias and variance, improved robustness, substantially lower cost
Limitations	Prompt sensitivity, model-size dependence, bias favoring LLM-generated text	Limited scoring formats, indirect validation, unclear generalization beyond tested domains	Task-specific validation, unresolved panel selection strategy, untested performance on reasoning-heavy or clinical domains

## Data Availability

No new data were created or analyzed in this study. Data sharing is not applicable to this article.

## Abbreviations

The following abbreviations are used in this manuscript:

AI	Artificial Intelligence
LLM	Large Language Model
LLM-as-a-Judge	Large Language Model as a Judge
EHR	Electronic Health Record
BLEU	Bilingual Evaluation Understudy
ROUGE	Recall-Oriented Understudy for Gisting Evaluation
BERTScore	Bidirectional Encoder Representations from Transformers Scoring
CoT	Chain-of-Thought
G-EVAL	Generative Evaluation Architecture using LLMs
PDSQI-9	Provider Documentation Summarization Quality Instrument (9-item)
ICC	Intraclass Correlation Coefficient
RAG	Retrieval-Augmented Generation
BHC	Brief Hospital Course
SOAP	Subjective, Objective, Assessment, Plan
GENMOD	General Model for Multi-Section Note Generation
SPECMOD	Specialized Model for Independent Section Generation
κ (Kappa)	Cohen’s Kappa Statistic
QA	Question Answering
CDC	Centers for Disease Control and Prevention
MedQuAD	Medical Question Answering Dataset
WAI-O-S	Working Alliance Inventory–Observer Short Form
ORS	Outcome Rating Scale
PoLL	Panel of LLM Evaluators
F1 Score	Harmonic Mean of Precision and Recall
